# Molecular regulation of estrogen receptors and recent advances on ERα36 splice variant in prostate cancer

**DOI:** 10.1210/jendso/bvag170

**Published:** 2026-08-05

**Authors:** Deborah Simão Souza, Camila Rebelo Morgado Costa, Larissa Thainá Brito Dias, Giovanna Martins Gonçalves, Catarina Segreti Porto, Carolina Meloni Vicente

**Affiliations:** Laboratory of Experimental Endocrinology, Department of Pharmacology, Escola Paulista de Medicina (EPM), Universidade Federal de São Paulo (UNIFESP), São Paulo, SP 04039-032, Brazil; Laboratory of Experimental Endocrinology, Department of Pharmacology, Escola Paulista de Medicina (EPM), Universidade Federal de São Paulo (UNIFESP), São Paulo, SP 04039-032, Brazil; Laboratory of Experimental Endocrinology, Department of Pharmacology, Escola Paulista de Medicina (EPM), Universidade Federal de São Paulo (UNIFESP), São Paulo, SP 04039-032, Brazil; Laboratory of Experimental Endocrinology, Department of Pharmacology, Escola Paulista de Medicina (EPM), Universidade Federal de São Paulo (UNIFESP), São Paulo, SP 04039-032, Brazil; Laboratory of Experimental Endocrinology, Department of Pharmacology, Escola Paulista de Medicina (EPM), Universidade Federal de São Paulo (UNIFESP), São Paulo, SP 04039-032, Brazil; Laboratory of Experimental Endocrinology, Department of Pharmacology, Escola Paulista de Medicina (EPM), Universidade Federal de São Paulo (UNIFESP), São Paulo, SP 04039-032, Brazil

**Keywords:** estrogen receptor, ERα36 splice variant, prostate cancer cells

## Abstract

Prostate cancer is the second most common cause of cancer in men worldwide, and first-line therapies for metastatic disease include androgen deprivation therapy and androgen receptor pathway inhibitors, in addition to chemotherapy for selected patients. Although these strategies improve patient prognosis, progression to castration-resistant prostate cancer and therapeutic resistance remain clinically significant. In this review, we will explore published and new evidence of the expression and function of estrogen receptors (ERs) ERα and ERβ, as well as their variants, and the G protein-coupled estrogen receptor (GPER) in normal prostate gland, during development and adulthood and in prostate cancer cell lines. Consistent with these findings, studies from the literature have demonstrated that ERα, ERβ, and GPER are expressed in a cell-specific manner in the normal prostate gland. In the androgen-independent prostate cancer cells PC-3 (derived from bone metastasis) and DU-145 (brain metastasis), used in vitro and in xenograft implants as castration-resistant prostate cancer models, ERα and ERβ are present at transcript and protein levels, as well as the GPER. Importantly the presence of ERα36 splice variant is also detected in prostate cancer cells. Although the exact contributions of these modifications to prostate cancer advancement and treatment outcomes remain under investigation, the absence of selective inhibitors for the resulting ER variant proteins restricts deeper inquiry. Developing such targeted agents is crucial to uncovering variant biology in both healthy and malignant tissues, evaluating their therapeutic value in preclinical settings, and ultimately guiding clinical management.

Prostate cancer is the second most common cancer in men worldwide, with an estimated 1 466 680 cases and 396 792 deaths in 2022 (1, 2). Most cases (≥99%) are adenocarcinomas (3), and the median age at diagnosis is 67 years. Primary neuroendocrine prostate cancers, including small-cell variants, are rare (4). Approximately 75% of patients are diagnosed with disease confined to the prostate, a stage associated with a 5-year survival rate of 100%. Management at this stage typically involves active surveillance, prostatectomy, or radiation therapy, depending on the risk of progression. In contrast, about 10% of patients present with metastatic disease at diagnosis, for which the 5-year survival rate drops to 37%. Standard first-line treatments for metastatic prostate cancer include androgen deprivation therapy (ADT), via orchiectomy or administration of a gonadotropin-releasing hormone agonist (eg, leuprolide) or antagonist (eg, degarelix); androgen receptor pathway inhibitors, via androgen receptor inhibitors (eg, enzalutamide, darolutamide, apalutamide) or inhibitors of androgen biosynthesis (abiraterone) and, for selected patients, chemotherapy (docetaxel, cabazitaxel) (reviewed in 5).

The ADT and androgen receptor pathway inhibitors efficiencies are restricted by the functional consequence of androgen receptor (AR) point mutations and improved expression of constitutively active receptors. Numerous coactivators (>280) have been shown to interact with different regions of AR, and their functional impact has been examined under varying concentrations of androgens or antiandrogens. These coactivators are broadly expressed in prostate cancer and play an important role in ligand-dependent AR activation in models that represent both therapy sensitive and therapy-resistant cell lines (reviewed in 6, 7). In addition, the involvement of the glucocorticoid receptor in endocrine therapy-resistant prostate cancer has been reported, and distinct coactivators may also associate with this receptor, thereby contributing to therapy failure (reviewed in 6). Several reviews have discussed these issues in detail, but the development of castration-resistant prostate cancer (CRPC) and the resistance to treatment remain significant (reviewed in 5, 8, 9). This review addresses the role of estrogen receptors (ERα and ERβ), as well as their variants, and G protein-coupled estrogen receptor (GPER) on normal and prostate cancer with reference to ERα36 splice variant in prostate cancer cells.

## Overview of estrogen receptors: structural diversity and signaling pathways

ERα (encoded by *ESR1,* 6q25.1) and ERβ (encoded by *ESR2,* 14q23.2) are modular proteins, composed of 3 functional domains: the NH2-terminal domain, the DNA-binding domain, and the COOH-terminal ligand-binding domain (LBD) (reviewed in 10-12). The NH2-terminal domain contains a ligand-independent activation function domain that is required for transcriptional activation of target genes and shows only 16% sequence similarity between ERα and ERβ. The DNA-binding domain is highly conserved, sharing 97% amino acid identity between ERα and ERβ and is responsible for sequence-specific interaction of ERs with estrogen-responsive elements (EREs), in regulatory DNA sequences of target genes. ER dimers bind to ERE, which consist of an inverted repeat sequence separated by 3 nonspecific nucleotides, 5′-GGTCAnnnTGACC-3′, and sequences containing 1 or more deviations from this consensus motif. Upon binding of 17β-estradiol (E2), the E2-ER complex recruits co-regulatory proteins, facilitating chromatin remodeling and interactions with general transcription machinery leading to the activation of gene transcription (13, 14). ERs can interact with other transcription factor complexes, for example, Fos/Jun (AP-1-responsive elements) or SP-1 (GC-rich SP-1 motifs) and influence transcription of genes whose promoters do not harbor EREs. By comparison, the LBDs of ERα and ERβ share approximately 59% overall amino acid sequence identity, although their ligand-binding pockets differ only subtly in structure. Notably, these minor structural variations in the ligand-binding pockets have enabled the development of ligands with subtype selectivity. Propyl pyrazole triol (PPT) and 2, 3-bis (4-hydroxyphenyl)-propionitrile (DPN) are widely used agonists that selectively activate ERα and ERβ, respectively (15, 16). The LBD also contains a ligand-dependent activation domain (AF2) (reviewed in 10-12). In addition to the well-studied transcriptional effects of E2, there are rapid effects occurring within seconds or minutes after addition of E2. Ligand-activated membrane ERs initiate signaling by activating the nonreceptor tyrosine kinase (SRC) or via G-protein coupling, leading to the activation of kinase cascades including MAPK, phosphatidylinositol-3-kinase/serine threonine kinase (PI3K/AKT), cAMP-dependent protein kinase, and AMP-activated protein kinase (reviewed in 17). In different cell types, nuclear and membrane/cytoplasmic pools of ERα and ERβ have shown similar affinity for binding with E2. Membrane-initiated rapid signaling and classical transcriptional nuclear properties by ERs may function either separately as distinct pathways, or together as a fully integrated network (reviewed in 17, 18).

ESR1 (ERα) and ESR2 (ERβ) exhibit diverse alternatively spliced mRNA variants, with ERα (ERα66, ERα46, ERα36, ERα30, ERαi34, and ERα-LBD37.3) and ERβ (ERβ1, full-length, ERβ2, ERβ3, ERβ4, ERβ5, and ERβ6) having 6 proteins (reviewed in 19-21).

In 1996, an orphan 7-transmembrane-spanning G protein–coupled receptor termed GPR30, was first cloned (reviewed in 22). Subsequently, in 2007, GPR30 was officially renamed GPER (encoded by *GPER1*, 7p22.3) by the International Union of Basic and Clinical Pharmacology (reviewed in 23). GPER-mediated intracellular signaling is initiated through multiple heterotrimeric G proteins, including Gαs and Gαi, as well as Gβγ-dependent pathways. Moreover, GPER activation triggers transactivation of the epidermal growth factor receptor (EGFR). This process involves Gβγ-driven activation of SRC, followed by recruitment of integrin α5β1 and matrix metalloproteinase-dependent release of heparan-binding EGF-like growth factor, which subsequently transactivates EGFR, and downstream signaling cascades such as extracellular signal-regulated kinase1 and 2 ERK1/2 and PI3 K/AKT (reviewed in 24, 25). Studies have supported the idea that GPER transcriptional activation occurs independently of ER. ER and GPER may collaboratively and complementarily contribute to carcinogenesis (reviewed in 24). Nevertheless, further complexity has been added by the fact that selective estrogen receptor modulators (SERMs) and selective estrogen receptor degraders (SERDs) function as GPER agonists (reviewed in 25, 26).

Due to its presence in prostate tissue, it is important to understand the expression, the mechanism of action and role of ERs and GPER on biology of prostate and prostate cancer.

## Estrogen receptors on prostate gland of rodents and humans

The prostate gland is derived embryologically from the endodermal urogenital sinus and its development, growth and function throughout life are under androgenic regulation through AR (27). DHT is the precursor of 5α-androstane 3β, 17βdiol (3β-Adiol). 3β-Adiol is the endogenous ligand of ERβ in the prostate and DHT/AR and 3β-Adiol/ERβ may control androgen-mediated growth of the prostate (28). On the other hand, mice with a deletion of the Cyp19a1 gene, an aromatase enzyme essential for estrogen biosynthesis, do not develop prostate cancer, despite showing increased androgen production (29). In a recent study, using in vitro and in vivo models of normal prostate and prostate cancer, single-cell RNA-sequencing studies demonstrated that estrogens partially mimic the transcriptional response of androgens and activate specific biological pathways associated with proliferation and metabolism (30).

It has long been documented that estrogens influence prostate growth, tissue homeostasis, and disease across lifespan, acting through multiple ERs, including ERα, Erβ, and GPER, which are expressed in a cell-specific manner within the prostate. During development, 17β-estradiol (E2) plays an essential physiologic role in regulating prostate branching morphogenesis and epithelial differentiation via ERα and ERβ, respectively (31-34). Experimental studies in rodents have demonstrated that ERα is expressed in the stromal compartment during early prostate, morphogenesis, with expression levels markedly decreasing at puberty, supporting a distinct role for ERα in prostate development (35, 36).

In murine prostates, with stromal cell-specific deletion of ERα, showed that in fibroblast ERα modulates branching morphogenesis whereas in smooth muscle ERα regulates stromal cell proliferation and extracellular matrix deposition (31, 32).

In humans, ERα is expressed in stromal cells during fetal development (37, 38). Furthermore, ERα have been identified in human prostatic epithelial stem and progenitor cells, where they facilitate estrogen-induced stimulation of stem cell self-renewal and progenitor cell proliferation (39, 40). These findings suggest a possible role for estrogens in maintaining prostate epithelial homeostasis by supporting the renewal of stem population. Estrogen effects within stem-progenitor cell pool are mediated through both classical genomic mechanism and membrane-initiated signaling pathways (41).

In contrast to ERα, primarily localized to stromal cells, ERβ is mostly found in prostatic epithelium with limited stromal expression in adult prostate. ERβ is well expressed in epithelial cells of the mouse and human prostate (42, 43). In developing rat prostate, ERβ expression is low at birth and increases as epithelial cells differentiate postnatally (33). ERβ knockout (ERβ^−/−^) mice demonstrate high proliferation in the epithelial cells with low-grade prostatic intraepithelial neoplasia and inflammatory cell infiltration in the ventral prostate (44, 45). However, the ERβ knockout mice, created by removing DNA-binding domain of the ERβ gene, did not demonstrate the similar phenotypes (46). Comparison of the transcripts (by RNA-sequencing) of wild-type and interruption of the gene with a neocassette (Oliver Smithies ERβ knockout mice [ERβ^OS−/−^]) showed that genes involved in prostate cancer were increased upon inactivation of ERβ (47). Despite its regulation of genes associated with prostate cancer, ERβ^OS−/−^ mice do not develop prostate cancer. ERβ knockout mice by using CRISPR/Cas9 technology, ERβ^crispr−/−^ mice, develop epithelial hyperplasia and intraductal cancer-like lesions in the ventral prostate (48). The phenotype of the ventral prostate in ERβ^crispr−/−^ mice was similar to, but more severe than, that in the ERβ^OS−/−^ mice. In the ventral prostate of 6-month-old ERβ^crispr−/−^ mice there was epithelial hyperplasia, fibroplasia, inflammation, stromal overgrowth, and intraductal cancer-like lesions. This was accompanied by an increase in Ki67 and P63 and loss in DACH1 and PURα, 2 AR repressors (48). A recent study comparing RNA-sequencing of ERβ^crispr−/−^ and wild-type mice demonstrated that basal cell genes were significantly upregulated with abnormal basal cells expressing P63 and AR in the ERβ^crispr−/−^ mouse prostates. Testosterone treatment significantly inhibited prostatic inflammation in ERβ^crispr−/−^ mice, suggesting a role of ERβ in maintaining the normal basal cells and in modulating the immune environment in mouse prostate (49).

In human fetal prostate, ERβ is broadly expressed in both epithelial and stromal compartments as early as week 7 of development and remains detectable throughout gestation and for several months after birth, supporting a role in prostate development (37, 38). In addition, ERβ is present in human prostate stem cells, where it limits symmetric self-renewal while favoring differentiation toward the progenitor lineage (39, 40). In the adult human prostate, reports on ERβ localization are inconsistent, with some studies describing expression restricted to basal epithelial cell (50, 51), whereas others report substantial levels in both basal and luminal epithelial layers (52, 53).

Finally, GPER was detected in mice prostate, suggesting it as the main mediator of estrogen action (54). A strong GPER immunoreactivity was observed in the cytoplasm of basal epithelial cells, whereas no immunostaining was observed in luminal secretory epithelial cells of human prostate gland. Furthermore, a weak staining in the cytoplasm of stromal cells was also detected (55).

It is worth mentioning that both murine and human prostates are also composed of an outer layer of luminal epithelial cells, lining the duct that expresses high level of AR, and an inner layer of AR-negative basal cells (56). Previous studies have shown that subsets of basal cells in mouse prostate contain multipotent stem cells, which can be the original cells of prostate cancer (57-59).

Even though these studies have demonstrated the importance of ER and AR in prostate biology, additional studies are required to fully understand the ER-AR relationship in prostate biology and determine how ER can effectively be targeted to avoid or overcome treatment resistance.

## Expression of the classical estrogen receptors ERα and ERβ in prostate cancer tissue samples and cell lines

Estrogen signaling is associated with prostate cancer progression (reviewed in 60-62). Plasma 17β-estradiol (E2) levels are positively correlated with high-grade prostate cancer (63) and in patients undergoing ADTs with evolution to CRPC (64). The effects of estrogens on prostate cells are thought to be mostly mediated by the classical ERs, either through ligand-activated transcriptional regulation (genomic pathway) or by triggering cytoplasmic-signaling cascades (rapid action or nongenomic pathway) (65, reviewed in 61, 66-68). Furthermore, GPER, which activates a diverse array of signaling pathways (reviewed in 23, 24), also play a role in prostate cancer cells.

## A. Estrogen receptors in prostate cancer tissue samples

High-grade prostatic intraepithelial neoplasia showed ERα mRNA and protein expression in 28% and 11% of cases evaluated. ERα is detected in a minority of low- to intermediate-grade adenocarcinoma. High-grade (primary Gleason grade 4 and 5) tumors revealed ERα expression in 43% of cases. The most significant ERα expression is observed in hormone refractory tumors and metastatic lesions, including lymph node and bone metastases (61, 69). A recent study, using 2 human prostate cancer tissue microarray cohorts, has shown that ERα expression in prostate cancer is heterogeneous among patients, ERα is detected in only half of the tumors. When expressed, it is associated with a more aggressive disease (30).

ERβ expression in human prostate epithelial cells declines during prostate cancer initiation and progression to higher-grade disease (50, 70-72) and loss of ERβ has been associated with induction of the epithelial-mesenchymal transition (73). Conversely, other studies have reported reexpression of ERβ as prostate cancer spreads to distant metastatic sites (52, 74). Elevated ERβ levels accompanied by increased cellular proliferation has also been observed in untreated hormone-naïve prostate tumors (75). Together, these observations indicate that ERβ may exert a protective function in the early stages of prostate carcinogenesis, while potentially facilitating metastatic progression at later stages of the disease.

## B. Estrogen receptors in prostate cancer cell lines

The androgen-dependent prostate cancer cells LNCaP (derived from lymph node metastasis) are AR-positive and show high dependency on androgens for growth. However, these cells are CRPC, because the patients from whom the primary cells were isolated showed a progression of the disease despite being treated with ADTs. Moreover, an *AR* mutation that confers resistance to castration is also observed in LNCaP cells (76). In these cells, mRNAs of ERα and ERβ were detected (77). The androgen-independent prostate cancer cells PC-3 (derived from bone metastasis) and DU-145 (derived from brain metastasis), do not express AR (78), and are used in vitro and in xenograft implants as CRPC models, ERα and ERβ are present at transcript (77, 79, 80) and protein levels (65, 80-82). On the other hand, 1 study has suggested that PC-3 and DU-145 cells did not express detectable protein levels of ERα and ERβ (83).

Determining the precise expression of ERs in tissues and cells remains challenging because of several technical variables (reviewed in 66, 84-86). Key issues include the use of nonspecific or poorly validated antibodies, as well as the sensitivity of immunohistochemical results in different fixation and processing, including antigen retrieval methods (reviewed in 87-89). Furthermore, ERβ splice variants, which have a modified C-terminal, will not be recognized by antibodies raised against the C-terminal peptide of ERβ. Therefore, antibodies raised against the N-terminal of ERβ are important for ERβ1 and all its splice variants, but the epitopes should not include threonine, serine, or tyrosine residues because these residues may be phosphorylated (reviewed in 89). Additionally, the inherent variability of immortalized cell lines across different laboratories and passage numbers further compounds these uncertainties (90). We suggest that the data should be interpreted carefully regarding the expression and localization of ERα and ERβ in human tissues and cell lines. Other methodologies, such as CRISPR/Cas9 technology, should be used to study the expression of these receptors in different cells of normal prostate, during development, and also in prostate cancer development.

Using validated antibodies (88), our research group established that although ERα and ERβ (ERβ1) are predominantly nuclear in human postpubertal prostate epithelial cells PNT1A and control cells (Sertoli and NT2/D1) (65, 91), they are largely localized in the extranuclear compartment of androgen-independent prostate cancer cells (PC-3 and DU-145) (65, 80). The nucleocytoplasmic transport of proteins is highly regulated and coordinated by exportin CRM1 (*Chromosome region maintenance 1*), also known as XPO1 (reviewed in 92, 93). E2-dependent interaction of ERα with CRM1 is shown in breast cancer cells (94). In DU-145 cells, the CRM1 inhibitor Leptomycin B significantly diminishes extranuclear ERα and ERβ levels while promoting their nuclear accumulation. This suggests that CRM1 plays a key role in the nucleocytoplasmic shuttling of estrogen receptors within these cells (65, 94). ERs localization are not static, but rather, dynamic with continuous shuttling between the nucleus and the cytoplasm (65). Interestingly, various stimuli such as growth factors, SERDs, SERMs, and 17β-estradiol regulate the bidirectional transport of ERs across the nuclear membrane. Because gene expression depends on nuclear accumulation, the export process serves as a key regulator of transcriptional strength and duration. This precise spatial partitioning maintains the necessary ER concentrations for both nuclear and extranuclear roles. Ultimately, the dynamic shuttling of ERs, driven by multiple signaling pathways and posttranslational modification, is fundamental to their overall cellular function. Thus, this cellular process may serve as the target of novel therapeutic strategies; however, it is also clear that more studies are required to elucidate the molecular basis of ER subcellular transport in prostate cancer cells and its role in, or its interconnection with, other mechanisms that influence its expression, abundance, and activity in the endocrine resistance of prostate cancer cells.

## Mutations and alternative splice isoforms of ERα in prostate cancer

Mechanisms of endocrine therapy resistance, mainly in breast carcinoma, have shown mutations, fusions, and truncations of ESR1 (ERα) and ESR2 (ERβ), alternative splice isoforms, heterodimers, and complex interactions with multiple signaling pathways of ERs, including signal transduction by other nuclear receptors (reviewed in 20). In prostate cancer, meta-analysis of ERα (*ESR1*) germline polymorphisms has shown polymorphisms of PvuII and XbaI in specific populations and associate with significant increase of the disease risk (95, 96). In the plasma of patients with advanced prostate cancer, *ESR1* mutations (E380Q, L536Q, Y537S, and D538G) are detected. Thirty-six patients were included in this analysis (n = 24 patients with CRPC within 12 months of commencing ADT and n = 12 who developed CRPC beyond 12 months). ESR1 mutations E380Q, L536Q, Y537S, and D538G were found in both groups, regardless of time to development of CRPC. These mutations were associated with bone metastasis (97, reviewed in 98). However, these studies are initial and need more investigations into the role of ESR1 mutations.

In 2005, a new ERα splice variant, called ERα36, was identified. The transactivation domains ligand-independent activation function 1 and 2, present in ERα, are not present in this variant, a truncated LBD and a unique C-terminal region of 27 amino acids are detected. ERα36 LBD retains its ligand-binding ability despite the truncation (99, 100). ERα36 possesses 3 potential myristoylation sites located near the N-terminus; this localization favors myristoylation, whereas the putative myristoylation sites in ERα66 are found further away from the N-terminus of ER-α66. It is possible that the myristoylation sites in ERα36 are well positioned to initiate membrane-initiated estrogen signaling (100). This variant is mainly located at the plasma membrane, where it acts as a key mediator of estrogen nongenomic signaling different types of cancers, such as renal cell carcinoma, papillary thyroid carcinoma, laryngeal carcinoma, endometrial carcinoma, hepatocarcinoma, gastric cancer, neuronal tumors (neuroblastoma and glioblastoma), and breast cancer (reviewed in 101, 102).

In control patients and patients with advanced prostate cancer, only 1 study is reported in the literature related to the detection of ERα36 splice variant. ERα36 is found at lower levels in samples from patients with prostate cancer compared to healthy control samples. The samples of all 42 patients with prostate cancer were included (n = 9 hormone-sensitive and n = 33 castration-resistant). Although splice variant ERα36 was frequently observed, no convincing associations with castration resistance were identified (97).

**Figure 1:**
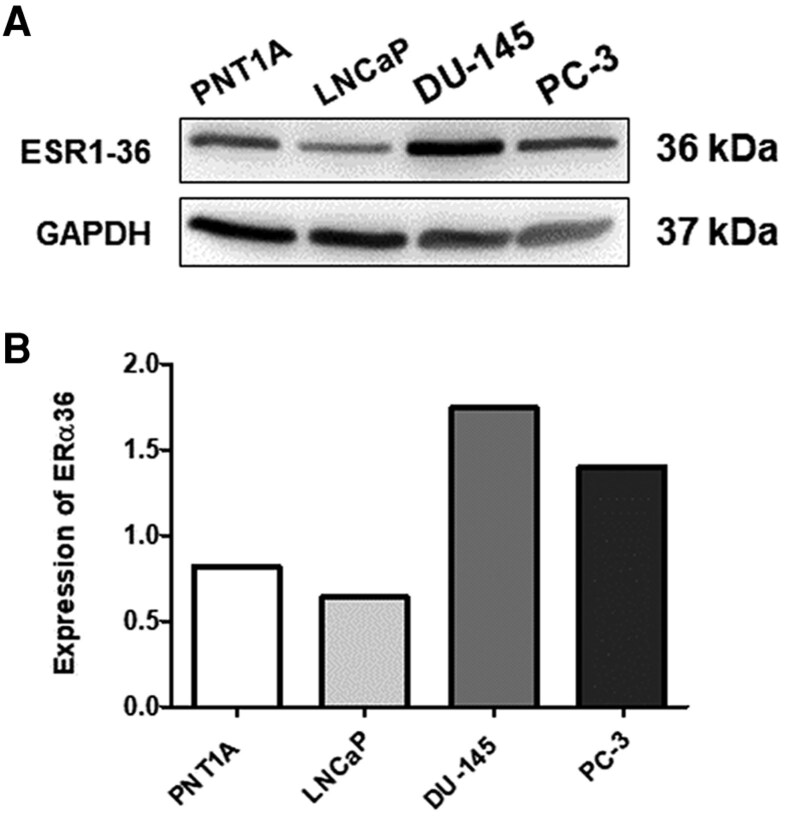
Expression of ERα36 in PNT1A, LNCaP, DU-145, and PC-3 cells. (A) The expression of ERα36 was determined by Western blot (as previously described in 80), using 50 μg of protein/lane and antibody specific for ERα36 against the carboxy-terminal region of ERα36 (Cell Applications, Cat# CY1109, discontinued, no RRID available) (top row) or antibody that recognizes GAPDH (Cell Signaling Technology Cat# 2118, RRID:AB_561053) (bottom panel). The protein sizes of ERα36 and GAPDH are shown on the right. (B) Bars represent the densitometric analysis of the Western blot assay. Results were normalized to GAPDH expression in each sample and plotted. The data shown are representative of 2 independent experiments.

The presence of ERα36 was shown in androgen-independent prostate cancer cells PC-3, by our laboratory. This endogenously synthesized ERα36 is not regulated by ERα, Erβ, or GPER activation (80). We present now unpublished results related to the protein levels of ERα36 in human post pubertal prostate epithelial cells PNT1A, androgen-dependent prostate cancer cells LNCaP, and androgen-independent prostate cancer cells DU-145 and also PC-3 (Fig. 1). All antibodies used in this manuscript are listed in Table 1. The expression is higher in DU-145 and PC-3 cells than in LNCaP and PNT1A (Fig. 1), suggesting that ARs may play a role in the regulation of the expression of ERα36. The interaction between the AR and ER splicing events is an emerging topic in cancer research, especially in breast and prostate cancer. Although the AR is traditionally known as a transcription factor, evidence indicates that it also acts as a regulator of mRNA processing, influencing the production of different protein isoforms. The ability of AR to induce splicing variants (such as AR variant, AR-V7, or ER variants) allows cancer cells to survive androgen deprivation therapies, making the tumor “castration-resistant.” Understanding how AR modulates ER splicing opens avenues for combination therapies that use splicing inhibitors along with traditional hormonal blockers.

**Table 1:** Antibodies used in the manuscript

Antibody	Identification
ERα36	Cell Applications, Cat# CY1109, discontinued, no RRID available, donated by Dr. Zhao-Yi Wang
GAPDH	Cell Signaling Technology Cat# 2118, RRID:AB_561053
GPER	Abcam Cat# ab39742, RRID:AB_1141090

**Figure 2:**
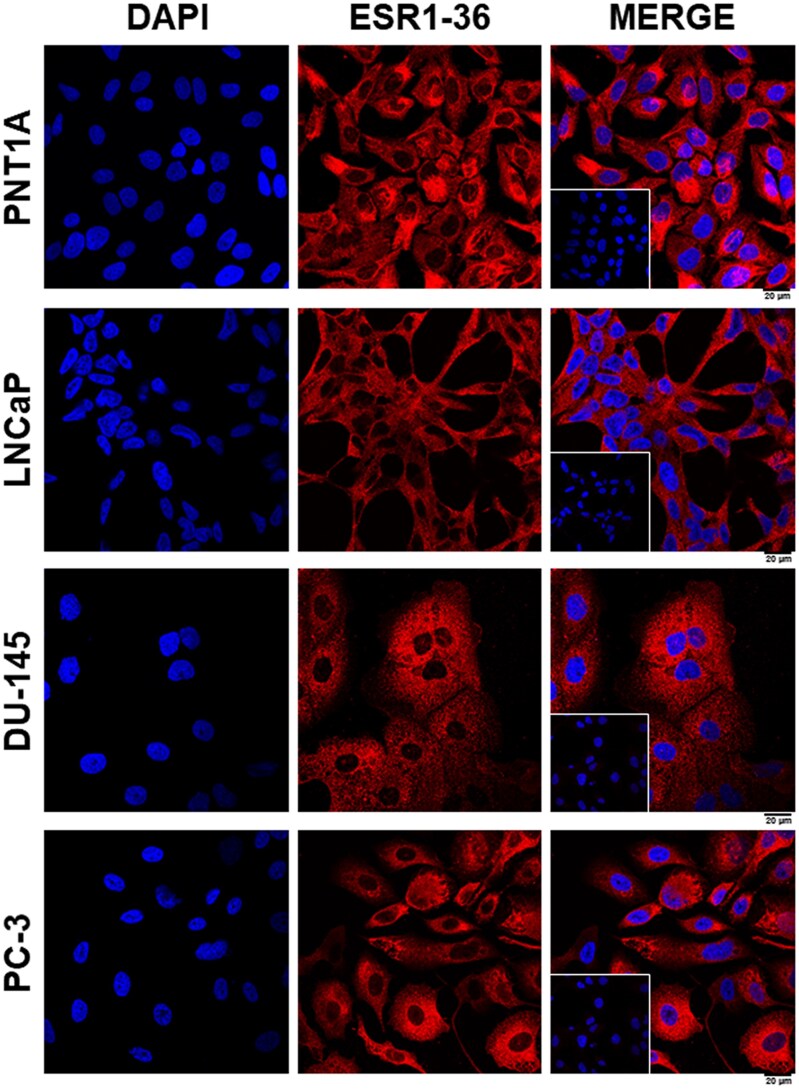
Immunofluorescence analysis of ERα36 in PNT1A, LNCaP, DU-145, and PC-3 cells. Immunostaining (red) for ERα36 was detected as previously described (80), using the same antibody indicated above (top panel, Fig. 1) and Alexa Fluor 594-labeled secondary antibody. Nuclei were stained with DAPI (blue). Negative control was performed in the absence of primary antibody (insert). Scale bar as indicated, 20 μm. The data shown are representative of 3 independent experiments.

The immunostaining of ERα36 is detected in cytoplasm and nuclei of the PNT1A cells, and this protein is mostly found in the cytoplasm of LNCaP, DU-145, and PC-3 cells (Fig. 2). In PC-3 cells, E2 induces phosphorylation of ERK1/2. This effect is not blocked by pretreatment of the cells with ERα- or ERβ-selective antagonists, or GPER-selective antagonist, or all 3 selective-antagonists together, suggesting that ERα36 may be involved in phosphorylation of ERK1/2 (80). This involvement need to be better investigated using other technologies, such as CRISPR/Cas9 and siRNA.

Multiple downstream pathways are modulated by E2-ERα36 within the breast cancer microenvironment. Through the SRC complex, it initiates paxillin-dependent cyclin D1 expression. ERα36 further sustains ERK2 signaling by physically interacting with the kinase to prevent dephosphorylation by MKP3 (103). Additionally, ERα36-mediated phosphorylation of EGFR (Y845) leads to subsequent ERK1/2 activation (104). Beyond these, the activation of PLC/PKC and PI3K/AKT signaling by E2-ERα36 regulates anti-apoptotic responses and metastatic progression (105, 106). Finally, tamoxifen has been shown to bind ERα36 directly, stimulating ALDH1A1 expression to enhance tumor stemness (107, reviewed in 101, 102).

These multiples signaling pathways and the role of ERα36, observed in breast cancer cells, need to be further investigated in prostate cancer. Furthermore, other isoforms (ERα46, ERα30, ERαi34, and ERα-LBD-37.3 kDa) need to be explored. The presence of the ERα36 in advanced prostate cancer tissue samples and cell lines point out additional mechanisms beyond hotspot mutations that may drive prostate cancer progression and therapy resistance.

Currently, targeted therapies for specific ERα variants are nonexistent. This may be due to several reasons: (1) known ESR1 gene alterations and splice variants often enhance the sensitivity of breast cancer cells to standard treatments like tamoxifen and fulvestrant; (2) there is insufficient evidence confirming that many ERα variant mRNAs translate into proteins that significantly contribute to clinical drug resistance; and (3) the few ERα variants that have been clearly shown to be translated into proteins that drive therapy resistance were identified relatively recently, and the field is still trying to identify strategies for developing specific inhibitors. Consequently, further research in prostate cancers is essential to advance both prognostic and therapeutic outcomes.

## Mutations and alternative splice isoforms of ERβ in prostate cancer

The role of ESR2 mutations in prostate cancer is still not well understood and warrants further investigation. In patients with CRPC, 4 cases have been reported carrying the H92Y (c.274C > T) missense mutation (108, 109). In benign prostate glands, ERβ1 is localized principally in the nuclei of basal epithelial cells and some stromal cells. In contrast, ERβ2 and ERβ5 are localized in the cytoplasm of both basal and luminal epithelial cells. In specimens from a cohort of patients with prostate cancer, ERβ1 is lost in both nuclear and cytoplasmic compartments, but some remained. ERβ2 and ERβ5 display a diffused pattern of cytoplasmic, with occasional nuclear positivity (110). On the other hand, increase in the expression of cytoplasmic ERβ1 and nuclear ERβ2 has been associated with worse cancer-specific outcomes following radical prostatectomy (111). Another study confirms the presence of ERβ1, ERβ2, and ERβ5 and has shown the presence of ERβ4 in human prostate (5 control samples) (97). In the plasma of patients with advanced prostate cancer, ERβ (ERβ1) is detected at a significantly lower concentration than ERβ2, ERβ4, and ERβ5 (97). Additional studies are required to fully understand the expression and role of ERβ splice variant on prostate cancer development and CRPC.

## Estrogen receptor heterodimers in cancer

In breast cancer, ERα and ERβ can assemble into homodimers (ERα/ERα or ERβ/ERβ) or heterodimers (ERα/ERβ), and both forms can bind EREs in response to ligand binding (112). These different dimer configurations confer distinct functional properties. In addition, the progesterone receptor and AR are involved in complex crosstalk with ERα and may also form heterodimers that exhibit distinct functional properties (reviewed by 20).

In androgen-independent prostate DU-145 cells, colocalization of ERα and ERβ is detected mostly in the extranuclear region (65). Coimmunoprecipitation of ERα with ERβ in control (untreated DU-145 cells) and E2-treated cells is observed, indicating the presence of ERα/ERβ heterodimers in these cells. The activation of ERα/ERβ heterodimers in DU-145 cells by E2 induces the phosphorylation of ERK1/2 (65). Open questions remain concerning the dynamics between ERα or ERβ and their heterodimer partners. Specifically, the frequency and timing of these interactions, their transcriptional potential, and the identity of associated co-proteins remain poorly understood, as do their distinct functional contributions to both malignant and healthy prostate cells.

## Expression of GPER in prostate cancer

Immunohistochemistry and Western blotting have identified GPER expression in both healthy and malignant prostatic tissues. Although basal epithelial cells in benign prostates show robust GPER reactivity, luminal cells remain negative, and stromal cells exhibit only faint staining. Although present in adenocarcinomas, GPER levels decline as Gleason patterns progress from 2 to 4. Notably, intense GPER expression is also characteristic of high-grade prostatic intraepithelial neoplasia (55). In protein extracts of benign and neoplastic prostatic tissues, the presence of a GPER ∼42-kDa band is detected (55). GPER expression is significantly higher in CRPC than in androgen-sensitive prostate cancer clinical specimens. Using androgen-dependent prostate cancer cells LNCaP xenograft to model prostate cancer growth during androgen-sensitive vs castration-resistant phase, GPER-selective agonist G-1(1-[4-(6-bromobenzo[1,3]dioxol-5yl)-3a,4,5,9b-tetrahydro-3H-cyclopenta[c]quinolin-8-yl]-ethanone) (113), inhibits growth of castration-resistant but not androgen-sensitive tumors (114, reviewed in 115).

**Figure 3:**
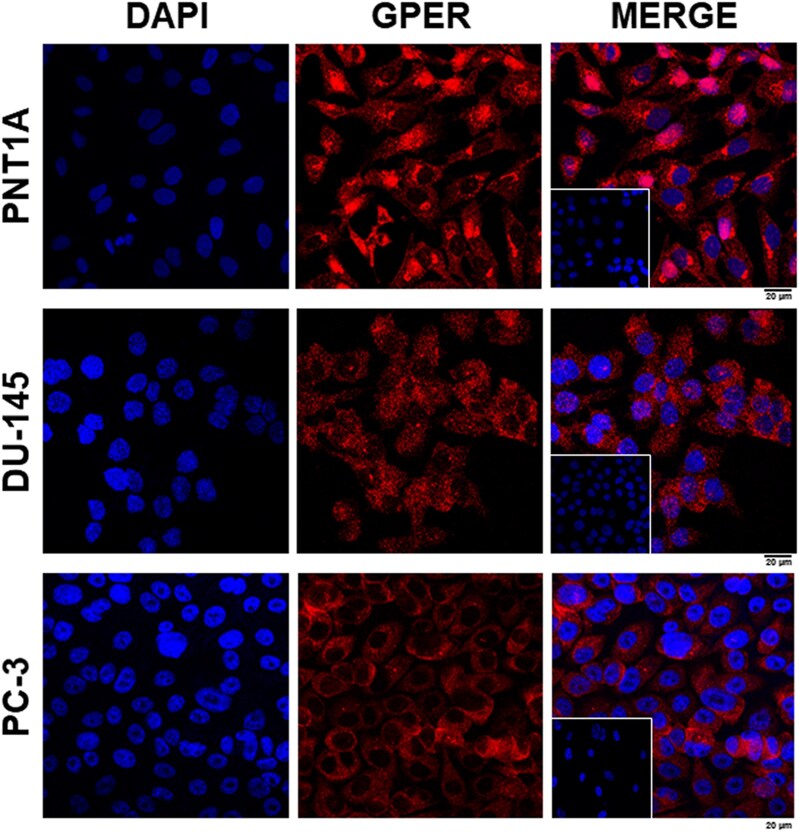
Immunofluorescence analysis of GPER in PNT1A, DU-145, and PC-3. Immunostaining (red) for GPER was detected previously described (80), using antibody specific for GPER (Abcam Cat# ab39742, RRID:AB_1141090) and Alexa Fluor 594-labeled secondary antibody. Nuclei were stained with DAPI (blue). Negative control was performed in the absence of primary antibody (insert). Scale bar as indicated, 20 μm. The data shown are representative of 3 independent experiments.

In the androgen-independent prostate cancer cells, the presence of the GPER (transcript and protein) is detected in PC-3 (80, 116) and DU-145 cells (transcript) (116). The transcript for GPER was higher in PC-3 than in DU-145 cells (116). We also observed the immunostaining of GPER in the cytoplasm and nuclei of the human postpubertal prostate epithelial cells PNT1A, and in the cytoplasm of the androgen-independent prostate cancer cells DU-145 (Fig. 3) and PC-3 (80 and Fig. 3). In the majority of cell types, GPER is primarily observed within intracellular membranes, though its presence on the cell surface is modulated by steroid hormones and during tissue injury (reviewed in 117). Consistent with this, immunofluorescence imaging of various tissues, including healthy mammary and ovarian epithelia, as well as several cancer, reveals a predominantly internal staining profile (reviewed in 117). This localization is maintained by constitutive retrograde transport from the plasma membrane to the endoplasmic reticulum, a process potentially mediated by interactions with PDZ-binding proteins involved in receptor sorting (reviewed in 117).

## The role of ERα and ERβ, their isoforms, and GPER in prostate cancer

The complex interplay among ERα, ERβ, their various isoforms, and GPER has emerged as an important yet incompletely defined component of prostate cancer biology, influencing tumor progression, hormone responsiveness, and therapeutic resistance. In AR-positive LNCaP cells, ERβ activation down-regulates AR transcript, protein levels, and transcriptional activity, suggesting a tumor-suppressive role for ERβ in AR-positive prostate cancer cells (118). The activation of ERβ induced inhibition of cell proliferation and repression of MYC expression in LNCaP. The loss of ZFHX3 (the zinc-finger homeobox 3 (ZFHX3, also known as ATBF1 for AT motif-binding factor 1) prevented ERβ from suppressing cell proliferation and repressing MYC transcription. ZFHX3 as a factor for suppressor activity of ER β in LNCaP (119).

In the androgen-independent prostate cancer PC-3 (80) and DU-145 cells (65), the treatment with E2, ERα-selective agonist PPT or ERβ (ERβ1)-selective agonist DPN for 5 or 10 minutes increases the phosphorylation of ERK1/2. E2 also increases the phosphorylation of AKT (at Ser473) (120) and SRC (Tyr419) (120) in PC-3 cells. On the other hand, phosphorylated AKT was not detected in DU-145 cells (65).

In PC-3 cells, the E2-ERα/SRC or E2-ERβ/SRC complexes drive the expression of active, nonphosphorylated β-catenin (121), which subsequently triggers the upregulation of vascular endothelial growth factor A (VEGF-A) (122). The regulation of VEGF in both localized and metastatic prostate cancer involves several stages between transcription and translation. These mechanisms primarily include pre-mRNA alternative splicing, mRNA stability, and translational control (123). Conversely, Mak et al (2010) demonstrated that ERβ1, and its ligand 3β-Adiol, inhibits mesenchymal features in prostate cancer cells. This receptor promotes the destabilization of HIF-1α and downregulates the transcriptional activity of VEGF-A and TGFβ in PC-3 and LNCaP lineages. However, the precise transcriptional and post-transcriptional mechanisms by which ERs regulate VEGF in PC-3 cells remain to be fully elucidated.

**Figure 4:**
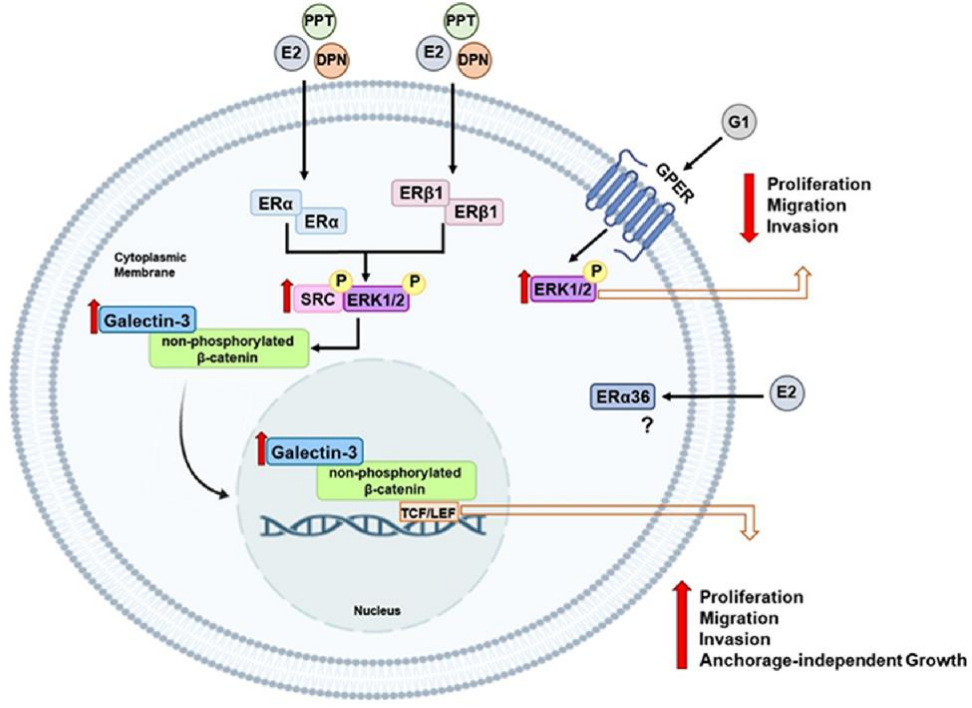
Illustration of the molecular regulatory mechanisms of estrogen receptor activations on androgen-independent prostate cancer cells PC-3 and DU-145. Activation of estrogen receptors ERα (E2 or PPT) and ERβ1 (E2 or DPN) increases the phosphorylation of SRC and ERK1/2 and the expression of Galectin-3 and nonphosphorylated β-catenin, which in turn can modulate nuclear transcriptional events, such as, proliferation, migration, invasion, and anchorage-independent growth of androgen-independent prostate cancer cells (see 65, 80-82, 120-122, 125, 126 for more information). Activation of G protein–coupled estrogen receptor (GPER) increases the phosphorylation of ERK1/2 and decreases proliferation, migration and invasion (80, 116, 127). ERα36 is present androgen-dependent and androgen-independent prostate cancer cells, and the role need to be determined. TCF/LEF (T-cell-specific transcription factor/lymphoid enhancer-binding factor).

The intracellular proteins SRC, PI3K/AKT, and the complex nonphosphorylated β-catenin/TCF/LEF (T-cell-specific transcription factor/lymphoid enhancer-binding factor) are involved in the enhance of the tumorigenic potential of PC-3 cells, increasing cell proliferation, migration, invasion, and tumor formation simulated by activation of ERα and/or ERβ (120-122) (illustration in Fig. 4). In fact, the cross-regulation of WNT/β-catenin/TCF/LEF signaling with members of the nuclear receptor family has been shown as a critical area of endocrine cell biology and cancer (reviewed in 124).

Nonphosphorylated β-catenin binds to Galectin-3 (GAL-3), a member of the β-galactoside-binding protein family (reviewed by 82, 128). GAL-3 interacts with various transcription factors to modulate gene expression linked to tumor plasticity (reviewed in 82, 129). Our studies demonstrated that ERα and ERβ contribute to the upregulation of both nonphosphorylated β-catenin and GAL-3 in PC-3 and DU-145 cells (81, 120, 125, 126), where these proteins primarily colocalize in the cytoplasm and occasionally in the nuclei (126). Furthermore, the GAL-3 inhibitor (VA03) prevents the ER-induced increase of nonphosphorylated β-catenin (81, 126), suggesting a functional hierarchy where GAL-3 acts upstream of nonphosphorylated β-catenin in DU-145 cells. Thus, ER activation enhances the expression, colocalization, and signaling of this pathway, with nonphosphorylated β-catenin serving as a downstream regulator of cell proliferation, migration, and invasion (81, 126) (illustration in Fig. 4). The activation of TCF/LEF by β-catenin also induces growth, migration, and invasion of other prostate cancer cells (130-132), collaborating with our studies. This may evolve into a promising area in the development of inhibitors targeting these proteins.

In advanced prostate cancer, the Wnt/β-catenin pathway is regularly turned on, driving the emergence of treatment resistance (reviewed in 133). Although primary tumors rarely exhibit activating mutations within this cascade, late-stage disease triggers it through an alternative route. These include growth factors and cytokines produced by the damaged tumor microenvironment, release of the coactivator β-catenin from sequestration after inhibition of AR signaling, altered expression of Wnt ligands and factors that modulate the Wnt signaling, and therapy-induced cellular senescence (reviewed in 134). Research on genetically engineered mice demonstrates that activating the Wnt/β-catenin pathway in the prostate drives oncogenesis and castrate-resistant growth (reviewed in 134). Furthermore, this activation triggers epithelial-to-mesenchymal transition, promotes neuroendocrine differentiation, and confers stem cell-like features to prostate cells (reviewed in 133, 134). The crosstalk of ER and Wnt/β-catenin signaling in prostate cancer initiation, development, or metastasis remain to be explored. These important roles of Wnt/β-catenin signaling in prostate cancer progression underscore the need for the development of drugs targeting this pathway to treat therapy-resistant prostate cancer.

In PC-3 and 22Rv1 androgen-independent prostate cancer cell lines transfected with either ERβ1 or ERβ2, when cultured in fetal calf serum, ERβ1 inhibited proliferation and factors known to be involved in bone metastasis whereas ERβ2 promoted proliferation, compared to nontransfected control cells (135). In addition, stable, ectopic expression of ERβ2 or ERβ5 enhanced PC-3 cell invasiveness but only PC-3 cells expressing ERβ5 exhibited augmented cell migration (110). ERβ1 and ERβ2 are differentially regulated during cell cycle in androgen-dependent prostate cancer LNCaP cells. Unlike ERβ2 which is expressed in G2/M phase, ERβ1 induces in response to E2 a cell-cycle arrest in early G1 phase through a nongenomic pathway involving c-Jun N-terminal kinase (136). It is important to mention that the expression and role of each ERβ isoform remain controversial in cell lines and during the progression of prostate cancer, and in patients with advanced and metastatic prostate cancer (reviewed in 66, 98, 102, 137).

In relation to GPER and prostate cancer, G-1 (GPER-selective agonist) induces cell growth-inhibition in both androgen-dependent (LNCaP) and androgen-independent (PC-3) cells in vitro and in vivo. The mechanisms underlying G-1-induced inhibition of PC-3 cell growth, are observed by a sustained activation of ERK1/2 and c-jun/c-fos-dependent upregulation of p21 (116; reviewed in 115) (illustration in Fig. 4). In fact, G-1 for 10 minutes increases the phosphorylation state of ERK1/2 in the PC-3 cells and this effect is blocked by pretreatment with its respective antagonists G15 (80). G-1 activates Gαi1 proteins to sustain ERK1/2 activation but not activate adenylyl cyclase (AC) for cAMP production in PC-3 cells. G-1 inhibits PC-3 cell migration and invasion, reduces the formations of filopodia and stress fibers (127). The treatment of G1 (1 µM and 1 nM) for 24 hours did not alter the expression of the ERα36 in PC-3 cells (80).

In prostate cancer, carcinoma-associated fibroblasts exhibit elevated GPER levels alongside reduced ERα expression. Moreover, GPER colocalizes with FAP, CD44, and nonmuscle myosin heavy chain B (SMemb). Experimentally, elevating GPER or silencing ERα in WPMY-1 human prostate stromal cells drives the upregulation of FAP, CD44, and SMemb, while decreasing smooth muscle markers. Additionally, media conditioned by these modified stromal cells accelerates the proliferation and migration of LNCaP and PC-3 lines. Conversely, GPER silencing or ERα upregulation reverses these outcomes. Mechanistically, GPER restricts ERα expression by suppressing the cAMP/protein kinase signaling pathway. Consequently, these findings highlight that GPER-mediated downregulation of ERα serves as a primary driver of prostate stromal cell activation (138). In cancer cell lines, including breast, endometrial, thyroid, and ovarian, G-1 promotes proliferation and associated signaling pathways. Differences in mechanisms of cellular proliferation as well as G-1 concentrations used may account for these in vitro differences (reviewed in 25). Furthermore, there is controversy as to whether GPER acts as an autonomous estrogen receptor in vivo, or whether ER and GPER may collaboratively and complementarily contribute to carcinogenesis (reviewed in 24). All these mechanisms need to be further investigated in prostate cancer.

## Therapies targeting estrogen receptors in prostate cancer

SERMs, such as tamoxifen and toremifene, have been studied in the prevention of prostate cancer. Several studies have attempted to evaluate the efficacy of SERMs in different clinical settings, such as treating high-grade prostate intraepithelial neoplasia, to prevent prostate cancer recurrence following surgery, treating naive bone metastatic prostate cancer, or treating CRPC. However, conflicting results were obtained, with either positive responses (139-141) or no significant changes (142-144). Tamoxifen treatment of the prostate cancer PC-3 cells induced apoptosis and inhibited the expression of ERα (145). Although these results have been achieved in vitro, tamoxifen has not provided a clinical benefit in prostate cancer patients in numerous clinical trials (reviewed in 146).

Another class of ER inhibitors are the SERDs, which includes the widely used drug fulvestrant. Treatment of androgen-dependent LNCaP cells with fulvestrant has been shown to decrease the expression of AR at the mRNA and protein level and to reduce the proliferation of prostate cancer cells in response to androgens with downregulation of AR in LNCaP (147) and also reduce the proliferation in androgen-independent PC-3 and DU-145 cells (148). Fulvestrant-regulated gene expression in DU-145 cells mediated by ERβ/NFκB crosstalk (149), and upregulation of hsa-miR-765 expression to suppress oncogenic HMGA1 (high-mobility group AT-hook) protein expression (150). In model of human prostate cancer cell line-resistant to enzalutamide (LNCAP-EnzaR), were detected tumor phenotypic changes, characteristic of an epithelial-mesenchymal switch; reduction of tumor susceptibility to natural killer (NK)-mediated lysis and upregulation of ERα expression (151). The treatment of these cells with fulvestrant restored the formation of target/NK cell conjugates and increased susceptibility to NK cell lysis. The activity of fulvestrant against LNCAP-EnzaR cells in vivo was evaluated in the context of NSG MHC-class I/II-deficient mice. Fulvestrant demonstrated antitumor activity against enzalutamide-resistant cells, an effect that was associated with activation of NK cells (151). Additional studies are required to fully understand the role of fulvestrant in CRPC. In a phase II clinical trial, fulvestrant monotherapy demonstrated safety in patients with hormone-refractory prostate cancer, although it yielded no clinical or prostate-specific antigen improvements (152). Clinical studies with these compounds must be designed keeping in mind that patients should be selected carefully based on their clinical status, specific molecular characteristics of the tumor, and previous therapies.

Utilization of novel preclinical models such as patient-derived organoids and patient-derived xenografts will offer a complex multicell type environment, essential to understand the role of ERs on prostate cancer development and to facilitate transition from basic science to clinical applications.

## Concluding remarks and future outlooks

Substantial efforts have been invested to study the roles of ERs in prostate cancer, which has illuminated a common occurrence of DNA level alterations in the ER genes, as well as a variety of *ESR1 and ESR2* mRNA splice variants. The precise roles of these variants in the progression and therapeutic responses of prostate cancer are still being elucidated. Growing evidence shows that ERs interact complexly, forming heterodimers like ERα/ERβ or pairing with other nuclear receptors. Advanced spatial and systems-level biomolecular tools will further clarify these links. Moreover, ER transcriptional coactivators help regulate key cellular processes, including proliferation, apoptosis, migration, and invasion. Although AR coactivator studies can predict biochemical and clinical recurrence, often being overexpressed, amplified, or mutated in CRPC, many also trigger AR-independent pathways that may be ER-mediated instead. Additionally, the potential convergence of genomic and rapid pathways on target genes offers an intriguing mechanism for how ER finely tunes gene expression in prostate cancer, a process requiring deeper investigation.

Future outlooks, such as, the role of various ERα and ERβ mutations, truncations, fusions, and variants in promoting oncogenic progression and metastasis in the tissue-specific microenvironments are important focus areas, as is detangling the web of interconnected signaling pathways and genomic regulators that interact with ERs. There is increasing evidence that ER has complex interactions, including formation of heterodimers, with other nuclear receptors, and new spatial and systems-level biomolecular tools will undoubtedly shed light on these connections. Using our knowledge of ER, we can design next-generation therapeutics with improved bioavailability, therapeutic index, administration, and efficacy while avoiding toxicities and selective pressures that lead to therapy resistance. Furthermore, the field lacks specific pharmacological agents for targeting the ER variant proteins, which will be important for understanding the biology of ERs in prostate and prostate cancer, therapeutic significance in preclinical models, and eventually clinical disease.

## Data Availability

Some or all datasets generated during and/or analyzed during the current study are not publicly available but are available from the corresponding author on reasonable request.

## Abbreviations

ADT: androgen deprivation therapy
AKT: serine threonine kinase
AR: androgen receptor
CRPC: castration-resistant prostate cancer
DPN: 2, 3-bis (4-hydroxyphenyl)-propionitrile
EGFR: epidermal growth factor receptor
ER: estrogen receptor
ERE: estrogen-responsive element
GAL-3: Galectin-3
GPER: G protein-coupled estrogen receptor
LBD: ligand-binding domain
NK: natural killer
PI3K: phosphatidylinositol-3-kinase
PPT: propyl pyrazole triol
SERD: selective estrogen receptor degrader
SERM: selective estrogen receptor modulator
SRC: nonreceptor tyrosine kinase
VEGF: vascular endothelial growth factor
ZFHX3: zinc-finger homeobox 3
